# Characterization of Local Products for Their Industrial Use: The Case of Italian Potato Cultivars Analyzed by Untargeted and Targeted Methodologies

**DOI:** 10.3390/foods9091216

**Published:** 2020-09-02

**Authors:** Cinzia Ingallina, Mattia Spano, Anatoly P. Sobolev, Cristina Esposito, Cristina Santarcangelo, Alessandra Baldi, Maria Daglia, Luisa Mannina

**Affiliations:** 1 Department of Chemistry and Technology of Drugs, Sapienza University of Rome, Piazzale Aldo Moro 5, 00185 Rome, Italy; cinzia.ingallina@uniroma1.it (C.I.); mattia.spano@uniroma1.it (M.S.); luisa.mannina@uniroma1.it (L.M.); 2 Institute for Biological Systems, Magnetic Resonance Laboratory “Segre-Capitani”, CNR, Via Salaria Km 29.300, 00015 Monterotondo (Rome), Italy; 3 Department of Pharmacy, University of Naples Federico II, 80138 Naples, Italy; cristina.esposito@unina.it (C.E.); cristina.santarcangelo@unina.it (C.S.); 4 Tefarco Innova, Parco Area delle Scienze 27/A-Campus, 43124 Parma, Italy; alessandra.baldi.alimenti@gmail.com; 5 International Research Center for Food Nutrition and Safety, Jiangsu University, Zhenjiang 212013, China

**Keywords:** *Solanum tuberosum* L., potato cultivars, metabolite profiling, NMR (Nuclear Magnetic Resonance), RP-HPLC-PDA-ESI-MSn (Reversed Phase High-Performance Liquid Chromatography with Photodiode Array Detector and Electrospray Ionization Mass Detector), industrial products

## Abstract

The chemical characterization of local Italian potato cultivars is reported to promote their preservation and use as high quality raw material in food industries. Twenty potato (*Solanum tuberosum* L.) cultivars from Piedmont and Liguria Italian regions were investigated using NMR (Nuclear Magnetic Resonance) and RP-HPLC-PDA-ESI-MSn (Reversed Phase High-Performance Liquid Chromatography with Photodiode Array Detector and Electrospray Ionization Mass Detector) methodologies. Water soluble and lipophilic metabolites were identified and quantified. With respect to literature data, a more complete ^1^H (protonic) spectral assignment of the aqueous potato extracts was reported, whereas the ^1^H NMR assignment of potato organic extracts was reported here for the first time. Phenolics resulted to be in high concentrations in the purple–blue colored Rouge des Flandres, Bergerac, Fleur Bleu, and Blue Star cultivars. Servane, Piatlina, and Malou showed the highest amount of galacturonic acid, a marker of pectin presence, whereas Jelly cultivar was characterized by high levels of monosaccharides. Roseval and Rubra Spes contained high levels of citric acid involved in the inhibition of the enzymatic browning in fresh-cut potato. High levels of the amino acids involved in the formation of pleasant-smell volatile compounds during potato cooking were detected in Rouge des Flandres, Blue Star, Bergerac, Roseval, and Ratte cultivars. These results suggest that each local cultivar is characterized by a proper chemical profile related to specific proprieties that can be useful to obtain high quality industrial products.

## 1. Introduction

Nowadays potatoes (*Solanum tuberosum* L., Solanaceae) represent one of the most widespread food crop in the world after rice, wheat, and corn [1] due to their adaptability to different climates and soils and to their low price. From a nutritional point of view, potatoes are low-fat foods (about 1% of lipids) rich in carbohydrates. The protein content (about 2.1%) is higher than the one found in other roots and tubers, such as manioc (*Manihot esculenta Crantz*, Euphorbiaceae), sweet potatoes (*Ipomoea batatas* (L.) Lam.), and yam (*Dioscorea alata* L., Dioscoreacea) [2]. Potatoes contain several minerals, vitamins, fibers, and bioactive compounds such as polyphenols and carotenoids, important for achieving or maintaining human well-being [3,4]. 

More in detail, the chemical composition of potatoes has been largely studied through different analytical techniques over the years, underlining the great importance of this foodstuff. In particular, chromatographic methodologies have been largely used for the determination of different classes of compounds such as phenolics, sugars, organic acids, amino acids, and lipids. Regarding phenolics, hydroxycinnamic acid derivatives have been largely studied, being the most abundant phenolics in potatoes [5,6,7]. Glucose, fructose, and sucrose have been quantified as the most abundant sugars in potatoes, whereas other sugars have been identified in lower concentrations [5,8,9]. Several organic acids have been detected in potatoes, with citric and malic acids being the most abundant [8,10]. Regarding amino acids, literature data have been focused mainly in the study of the amino acids involved in the Maillard reaction such as asparagine, glutamine, and proline [5,11,12]; however, other amino acids were detected in potatoes [13]. The lipid fraction of potatoes has been also studied and characterized by means of chromatographic methodologies. In particular, linoleic fatty acid has been found to be the most abundant fatty acid in potatoes, followed by linolenic and saturated acids [14,15,16]. Polar lipids were also identified [14,15].

The untargeted NMR methodology have been also applied for the chemical characterization of potatoes [17,18], allowing the identification of different classes of compounds namely sugars, amino acids, organic acids, phenolics, and other metabolites.

Potatoes are widely consumed in the human diet both as homemade products and industries products often included in main and side dishes. Potato products from industries includes French fries, potatoes flakes, frozen products, and potatoes starch and, due to the modern lifestyle, their request has rapidly grown over time [19]. 

Nowadays potatoes are cultivated in 160 countries with over 4000 known cultivars and a production of 368 million tonnes [20]. According to the Food and Agriculture Organization of the United Nations (FAO) data, in Italy, the annual potato production is of 1.3 millions of tonnes [21] being Tuscany, Emilia-Romagna, Puglia, Campania, Abruzzo, and Sicily regions the major producers. In many Italian regions, local potatoes are also cultivated and used only for the local consumption. Unfortunately, tuber variety and biodiversity tend to be lost due to the agronomic and commercial selection of only a few varieties. Another factor that contribute to the loss of crop biodiversity is the replacement of local varieties with high-yielding species [22]. Therefore, a limited number of potato varieties are cultivated and tubers coming from extensive productions, sometimes deceptively labelled as “Made in Italy”, are imported to satisfy the high potato request.

In this paper, the chemical profile of twenty traditional potato cultivars grown in two north Italian regions (Liguria and Piedmont) was investigated by means of NMR (Nuclear Magnetic Resonance) and RP-HPLC-PDA-ESI-MSn (Reversed Phase High-Performance Liquid Chromatography with Photodiode Array Detector and Electrospray Ionization Mass Detector)methodologies to promote their valorization and use as high quality raw material in food industries. 

## 2. Materials and Methods 

### 2.1. Chemicals and Solvents

Deuterated water (D_2_O) 99.97% D, methanol-D_4_ 99.80% D, chloroform-D 99.80% D + 0.03% Tetramethylsilane (TMS), and 3-(trimethylsilyl)-propionic-2,2,3,3-d_4_ acid sodium salt (TSP) were purchased from Euriso–Top (Saclay, France). Anhydrous sodium carbonate (Na_2_CO_3_), anhydrous potassium phosphate dibasic, anhydrous potassium phosphate monobasic and HPLC chemical standards (chlorogenic acid, caffeic acid, ferulic acid, gallic acid, and galacturonic acid) were purchased from Sigma–Aldrich (St. Louis, MO, USA). Methanol (HPLC-grade), chloroform (HPLC-grade), and glacial acetic acid were purchased from Carlo Erba Reagenti (Milan, Italy). Double-distilled water was obtained using a Millipore Milli-Q Plus water treatment system (Millipore Bedford Corp., Bedford, MA, USA).

### 2.2. Sampling

**Figure 1:**
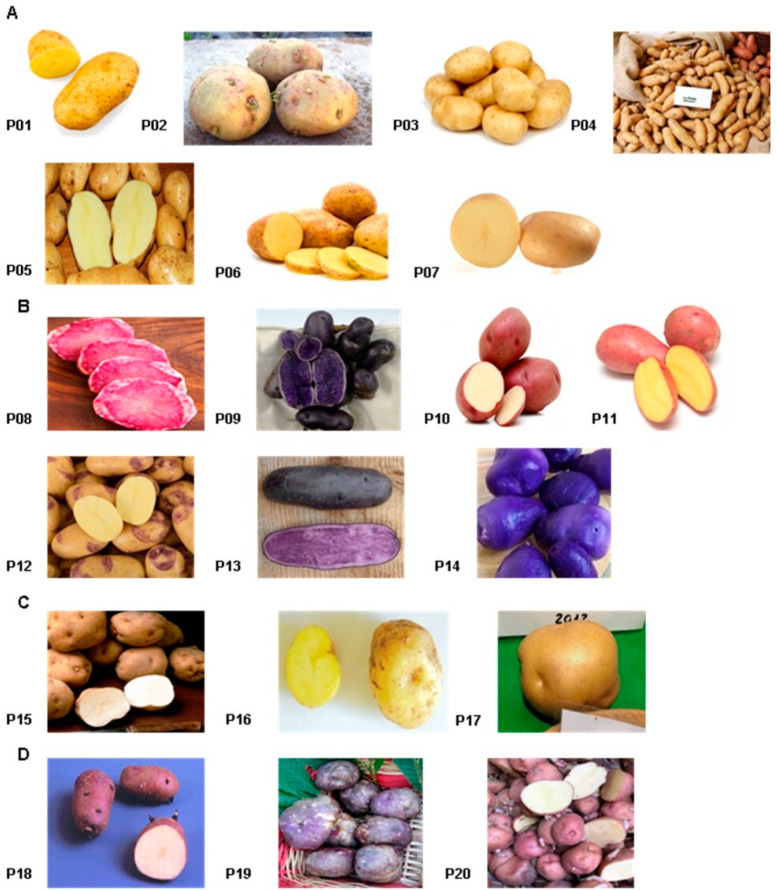
Piedmont cultivars characterized by yellow peel and yellow pulp (**A**) and colored peel and/or pulp (**B**). Liguria cultivars characterized by yellow peel and yellow pulp (**C**) and colored peel and yellow pulp (**D**).

Twenty potato cultivars (Figure 1) were produced and sampled by two farms, “Baclet Stefano” and “Consorzio della Quarantina”, located in two regions of northern Italy.

In particular, “Baclet Stefano” farm located in Pragelato area (Piedmont) provided fourteen varieties: Servane (P01), Piatlina (P02), Jelly (P03), Ratte (P04), Bintje (P05), Agria (P06), and Malou (P07) which are characterized by yellow peel and yellow pulp; Rouge des Flandres (P08), Bergerac (P09), Roseval (P10), Laura (P11), Blue Belle (P12), Fleur Bleu (P13), and Blue Star (P14) which are characterized by colored peel and/or pulp. “Consorzio della Quarantina” located in Torriglia (Liguria) provided six cultivars: Quarantina Bianca (P15), Giana Riunda (P16), and Gianita (P17) which are characterized by yellow peel and yellow pulp whereas Morella (P18), Quarantina Prugnona (P19), and Rubra Spes (P20) which are characterized by colored peel and yellow pulp. 

Tubers used for sowing were subjected to germination for four weeks in pots containing the same soil used for cultivation (third week of May 2018). Germination took place in rooms with controlled temperature (10 °C). Germinated tubers were then transplanted, during the last two weeks of June 2018, in sandy loam soil and common regional agronomic practices were applied (irrigation, nutrition, weed control, pest treatments). Plot design, same for both farms, is described in Appendix SA. Potatoes harvesting was carried out when tubers achieved their physiological maturity (first ten days of November). Then, potatoes were subjected to a curing period (during this period thickening of potato peel and slowing of the respiratory rate of the tubers occur, preparing them for storage) of 10 days at room temperature and finally stored at 4 °C until analysis. During the plant growth, the following monthly average value of temperature, humidity and rainfall were reported in Pragelato area (45°00′49″ N 6°56′29″ E): in July and August the mean temperature and humidity were of 24.5 °C and 65%, respectively, in September the mean temperature and humidity were of 20.3 °C and 71.6%, respectively, whereas in October the mean temperature and humidity were of 14.9 °C and 79.6%, respectively. During these months, no significative rainfall levels were detected. The following monthly average value of temperature, humidity, and rainfall were reported in Torriglia (44°31′03″ N 9°09′29″ E): in July, August and September the mean temperature and humidity were of 25.65 °C and 72.6%, respectively, whereas in September the mean temperature and humidity were of 18.8 °C and 63.6%, respectively. During these months, no significative rainfall levels were detected.

### 2.3. Sample Preparation

Tubers were cleaned with Na_2_CO_3_ aqueous solution to eliminate any external residual. For each cultivar, seven tubers were selected and each tuber was cut into eight pieces. One piece from each tuber was collected to have a representative sample consisting of seven random pieces for each cultivar. Samples were cut and chopped using a ceramic knife. After freeze-drying each cultivar sample was pulverized with mortar and pestle and stored in a cool and dry place until extraction.

### 2.4. Extraction Procedure for NMR Analysis

To prepare the samples to be submitted to NMR analysis, an extraction protocol previously described [23] was applied with some modifications. In details, 0.5 g of each sample was added sequentially to 3 mL of methanol/chloroform 2:1 *v/v* mixture, 1 mL of chloroform, and 1.8 mL of Millipore grade water. After each addition, the sample was carefully shaken. The emulsion was preserved at 4 °C for 40 min. Afterwards the sample was centrifuged (4200× *g* for 15 min at 4 °C) and the upper (hydroalcoholic) and lower (organic) phases were carefully separated. The pellets were subjected to a second extraction using half of the solvent volumes (in the same conditions described above) and the separated phases were pooled. The obtained extracts were dried using a soft N_2_ flow at room temperature until the solvent was completely evaporated. The dried extracts were stored at −20 °C until NMR analyses.

### 2.5. Extraction Procedure for RP-HPLC-PDA-ESI-MSn Analysis

For RP-HPLC-PDA-ESI-MSn analysis, the following extraction procedure was performed: 1 g of each sample was added to 10 mL of CH_3_OH/H_2_O/CH_3_COOH (70:29:1 *v/v/v*) mixture in a tube, carefully shaken for 1 min and sonicated for 15 min. To prevent the exposure to light, each tube was covered with an aluminum foil. Then, the sample was centrifuged at 5000× *g* for 5 min at 4 °C. The supernatant was separated from the pellet, which was extracted for a second time using the same procedure described above. The supernatants were collected and centrifuged at 5000× *g* for 5 min at 4 °C. The extract was filtered with PTFE (Polytetrafluoroethylene) filter (pore size 0.2 µm) prior to chromatographic analysis. One mL of extract was diluted 1:50 and filtered with PTFE filter (pore size 0.2 µm) prior to chromatographic analysis. When the concentration of some analyzed metabolite was over the maximum value of calibration curve, a greater dilution was made in order to bring the concentration back into the calibration range.

### 2.6. NMR Analysis for Metabolite Profile

The dried organic extract of each sample was dissolved in 0.7 mL of CDCl_3_/CD_3_OD (2:1 *v/v*) mixture and transferred into a 5 mm NMR tube that was then flamed-sealed. The dried hydroalcoholic extract was dissolved in 1 mL of D_2_O; 0.2 mL of this solution were diluted with 0.5 mL of 400 mM phosphate buffer in D_2_O (pH 7.4) containing 2 mM solution of trimethylsilylpropanoic acid (TSP) as internal standard and then transferred into a 5 mm NMR tube. NMR spectra of both organic and hydroalcoholic extracts were recorded at 28 °C on a Bruker AVANCE 600 spectrometer operating at the proton frequency of 600.13 MHz and equipped with a Bruker multinuclear z-gradient 5 mm probe head. ^1^H spectra (Bruker pulse sequence *zg*) of organic extracts were referenced to the residual CHD_2_ signal of methanol (3.31 ppm) and acquired with 128 transients, recycle delay of 5 s, acquisition time of 1.82 s, 90° pulse of 10–10.5 µs and 32 K data points. ^1^H spectra of hydroalcoholic extracts were referenced to methyl group signals of TSP (0.00 ppm) and acquired with 200 transients, recycle delay of 5 s, acquisition time of 2.28 s, 90° pulse of 14–14.5 µs, and 32 K data points. The residual HDO signal was suppressed using a pre-saturation (Bruker pulse sequence *zgpr*). The two-dimensional (2D) NMR experiments (^1^H-^1^H TOCSY (Total Correlated Spectroscopy), ^1^H-^13^C HSQC (Heteronuclear Single Quantum Correlation) and ^1^H-^13^C HMBC (Heteronuclear Multiple Bond Correlation)) were carried out under the same experimental conditions previously reported [24]. In order to evaluate the repeatability of the protocol, the complete procedure from the extraction to NMR measurement (for both hydroalcoholic and organic extracts) was repeated three times. The integrals of 26 selected signals in hydroalcoholic extract ^1^H NMR spectra, Supplementary Table S1, were measured using the Bruker TOPSPIN 1.3 software and normalized with respect to the methyl group signals of TSP, set to 100. The results have been expressed as mg/100 g (dry weight) ± standard deviation (SD). 

The integrals of eight selected signals in organic extract ^1^H NMR spectra, Supplementary Table S1, were measured using the Bruker TOPSPIN 1.3 software and normalized with respect to the resonance at 2.30 ppm, due to α-CH_2_ signal of total fatty acids, set to 100. The molar % values ± SD of fatty acids, sterol, phosphatidylethanolamine, phosphatidylcholine, and digalactosyldiacylglycerol have been calculated taking into account the number of equivalent protons using the following equations:

%_STE_ = 100(0.66I_STE_/I_tot_) (1)

%_TRI_ = 100(0.5I_TRI_/I_tot_) (2)

%_DI_ = 100(I_DI_/I_tot_) (3)

%_MONO_ = 100(I_UNS_ − 2I_DI_ − 1.5I_TRI_)/I_tot_ (4)

%_SAT_ = 100(I_FA_ − I_DI_ − 0.5I_TRI_ − %_MONO_)/I_tot_ (5)

%_PE_ = 100(2I_PE_/I_tot_) (6)

%_PC_ = 100(4I_PC_/9I_tot_) (7)

%_DGDG_ = 100(4I_DGDG_/I_tot_) (8)

where %_STE_, %_TRI_, %_DI_, %_MONO_, %_SAT_, %_PE_, %_PC_, and %_DGDG_ are molar % of β-sitosterol, tri-unsaturated fatty acids, di-unsaturated fatty acids, mono-unsaturated fatty acids, saturated fatty acids, phosphatidylethanolamine, phosphatidylcholine, and digalactosyldiacylglycerol, respectively. I_STE_, I_TRI_, I_DI_, I_UNS_, I_FA_, I_PE_, I_PC_, and I_DGDG_ are integrals, whereas I_tot_ is calculated according to the following equation:

I_tot_ = I_FA_ + 0.66I_STE_ (9)

### 2.7. Phenolic Content by RP-HPLC-PDA-ESI-MSn Analysis

The RP-HPLC-PDA-ESI-MSn analysis was performed using a Thermo Finnigan Surveyor Plus HPLC, equipped with a quaternary pump, a Surveyor UV-Vis diode array detector (DAD) and a LCQ Advantage Max ion trap mass spectrometer (Thermo Fisher Scientific, Waltham, MA, USA), connected through an ESI source. The autosampler operated at 4 °C and the column oven was maintained at 40 °C. The column was a Synergi Fusion RP-18 column (150 × 4.6 mm, 5 μm), with a Hypersil Gold C18 precolumn (10 × 2.1 mm, 5 μm), both sourced from Phenomenex (Torrance, CA, USA). The mobile phase was composed by eluent A 98% water, 2% methanol, 0.1% formic acid, 3 mM ammonium formate and eluent B 99% methanol, 0.1% formic acid, 3 mM ammonium formate. The elution gradient to separate the compounds was optimized as follows: 5% of B (0–0.2 min), 5–100% of B (0.2–11 min), 100% of B (11–12.2 min), and 5% of B (12.2–15 min). The injection volume was 0.5 μL and the flow equal to 0.45 mL/min. The total analysis time was 15 min per sample. Chromatogram measurements were taken at 280, 330, and 520 nm.

Spectral data were taken between 200 and 800 nm for every peak. HPLC-ESI-MSn data were obtained in both positive and negative ionization modes using the Xcalibur software. The ion trap was set to full scan (100–2000 *m/z*), data-dependent scan, and MSn modes. For the purposes of MSn data, collision energy was chosen to be 35% with an isolation bandwidth of 2 *m/z*. A preliminary experiment was executed for the purpose of optimizing MS operating parameters by using analytical standard solutions: 10 μg/mL gallic acid (50:50 *v/v*, 0.1% formic acid:methanol) and 10 μg/mL caffeic acid (50:50 *v/v*, 0.1% formic acid:methanol) solutions, infused through the ESI interface directly into the mass spectrometer, with a flow rate of 25 μL/min. Optimized parameters were set as follows: temperature 220 °C, spray voltage 4.5 and 5.0 kV, sheath gas 60, capillary auxiliary gas 20 and 25, capillary voltage −26.13 V and 35 V, for negative and positive ionization modes, respectively.

Results were expressed as mg/Kg (dry weight) ± SD. Three replications were made for each sample.

### 2.8. Statistical Analysis

Principal component analysis (PCA) and tree clustering analysis (TCA) were carried out on 37 selected variables. The 26 hydroalcoholic integrals present in Supplementary Table S1 were considered for statistical analysis. This number was reduced to 24 since glucose and galactose were expressed as the sum of their alpha and beta anomeric forms. Nine selected variables were due to the molar percentage of β-sitosterol, tri-unsaturated fatty acids, di-unsaturated fatty acids, mono-unsaturated fatty acids, total unsaturated fatty acids, total saturated fatty acids, phosphatidylethanolamine, phosphatidylcholine, and digalactosyldiacylglycerol. Four variables were due to the metabolites measured by chromatographic methodology. Gallic acid was excluded from statistical analysis since it was detected only in one cultivar. Before statistical analysis, the data were preprocessed using autoscaling: all the variables were mean-centered and each variable was divided by its standard deviation. Principal component analysis was carried out using SIMCA software (version 12), whereas tree clustering analysis was carried out using STATISTICA software (version 5.1). In Tree Clustering Analysis the city-block (Manhattan) distance, Equation (10), and the complete linkage method were used respectively as a measure of distance between samples and clusters [25].

distance (a, b) = Σ_i_ |x_ai_ − x_bi_| (10)

## 3. Results

Potato extracts from the 20 cultivars were analyzed by means of untargeted NMR spectroscopy to have a comprehensive metabolite profile of both hydroalcoholic and organic potato extracts and targeted RP-HPLC-PDA-ESI-MSn methodology focused on selected compounds (caffeic, chlorogenic, ferulic, gallic, and galacturonic acids).

The assignment of ^1^H NMR spectra of hydroalcoholic extracts was obtained by 2D NMR experiments (^1^H-^1^ TOCSY, ^1^H-^13^C HSQC and ^1^H-^13^C HMBC) and spiking experiments. Literature data regarding high resolution NMR [17,18] chemical assignment of potato extracts in different experimental conditions were used as references. With respect to literature data, a more complete ^1^H spectral assignment was obtained allowing the identification of fructose anomeric forms, galactose and myo-inositol, see Supplementary Table S2. In particular, both galactose anomeric forms were assigned by means of 2D experiments. The presence of β-galactose was suggested by its doublet (*J* = 8.0) at 4.60 ppm due to CH-1 axial proton. The diagnostic spin system detected by ^1^H-^1^H TOCSY experiment allowed to identify the other protons of this sugar, namely CH-2 (3.51 ppm), CH-3 (3.67 ppm), and CH-4 (3.5 ppm). Analogously, α-galactose was assigned by its doublet (*J* = 3.8 Hz) at 5.28 ppm and, through ^1^H-^1^H TOCSY experiment, CH-2 and CH-3 protons were assigned at 3.87 and 3.99 ppm, respectively. Myo-inositol was detected by its characteristic triplet (*J* = 9.5 Hz) at 3.30 ppm due to CH-4 proton. The diagnostic spin system detected by ^1^H-^1^H TOCSY experiment allowed to identify the protons at 3.57, 5.65, and 4.07 ppm, respectively, assigned to CH-2,5, CH-3,6, and CH-1. Fructofuranose was recognized by its characteristic multiplet at 4.12 ppm, belonged to CH-3 and CH-4 protons of beta anomeric form and to CH-3 proton of alpha anomeric form. The diagnostic spin system detected by ^1^H-^1^H TOCSY experiment allowed to identify protons at 3.81, 3.70, and 3.84 ppm of beta anomer CH-5 and CH-6,6’, respectively, and CH-5 proton at 4.07 of alpha anomer CH-5. β-Fructopyranose was recognized by its typical signal of CH-4 proton at 4.0 ppm. ^1^H-^1^H TOCSY experiment allowed to identify protons at 3.80, 4.04, 3.71, and 4.08 of CH-3, CH-5, and CH-6,6’, respectively.

**Figure 2:**
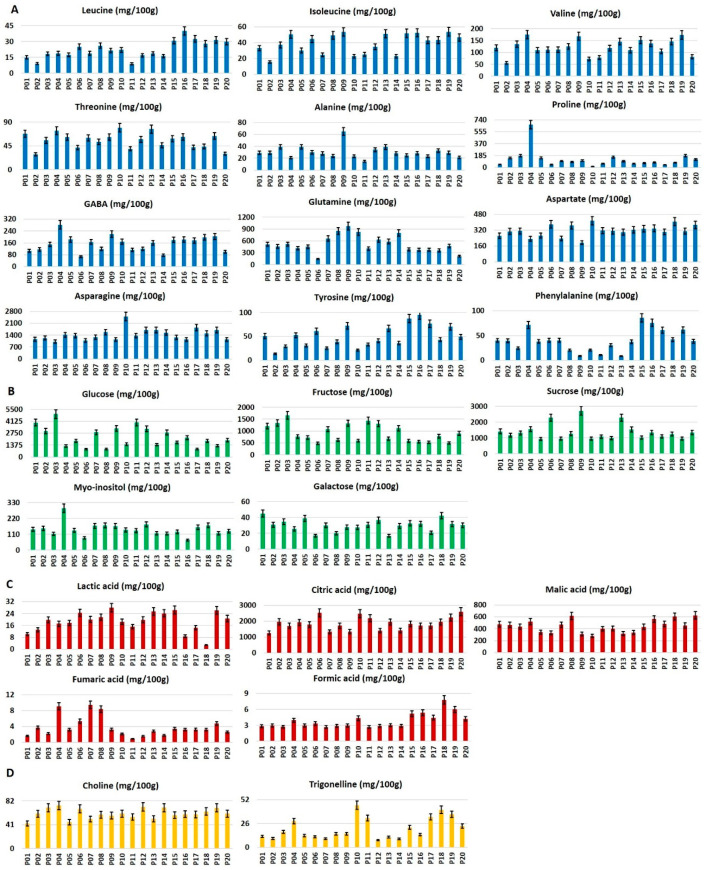
Histograms relative to metabolites (mg/100 g dry weight ± standard deviation SD) identified and quantified in NMR (Nuclear Magnetic Resonance) spectra of potato cultivars hydroalcoholic Bligh–Dyer extracts: (**A**) amino acids, (**B**) sugars, (**C**) organic acids, (**D**) other metabolites. Servane (P01), Piatlina (P02), Jelly (P03), Ratte (P04), Bintje (P05), Agria (P06) and Malou (P07), Rouge des Flandres (P08), Bergerac (P09), Roseval (P10), Laura (P11), Blue Belle (P12), Fleur Bleu (P13), Blue Star (P14), Quarantina Bianca (P15), Giana Riunda (P16), Gianita (P17), Morella (P18), Quarantina Prugnona (P19) and Rubra Spes (P20).

Five sugars, five organic acids, fifteen amino acids, two phenolics, choline, and trigonelline were identified in the hydroalcoholic extracts of all the analyzed samples. The compounds reported in Figure 2 were quantified. Arginine, glutamate, and lysine were not measured due to a strong signal overlapping.

**Figure 3:**
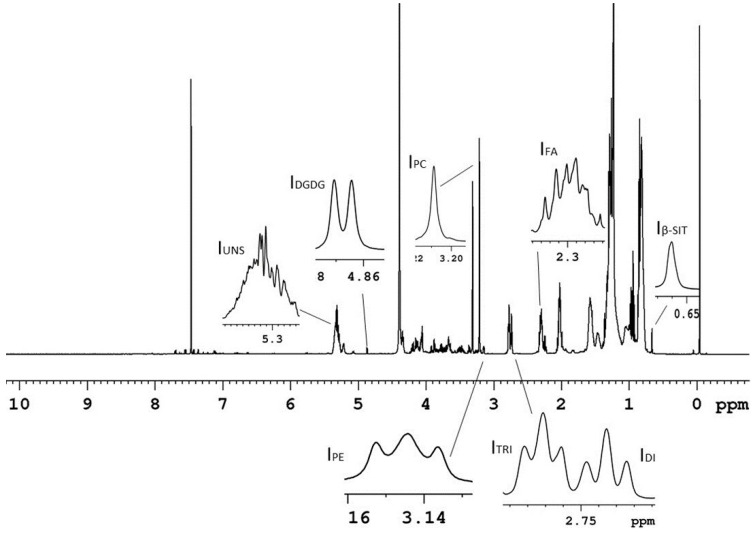
600 MHz ^1^H NMR spectrum (28 °C) of potato organic Bligh–Dyer extract dissolved in CDCl_3_/CD_3_OD (2:1 *v/v*) mixture. Signals used for the quantitative analysis were labelled: I**_β-SIT_**, CH_3_-18, β-sitosterol; I**_FA_**, CH_2_-2, all fatty acids; I**_DI_**, diallylic CH_2_, linoleic acid; I**_TRI_**, diallylic CH_2_, linolenic acid; I**_PE_**, CH_2_N, phosphatidylethanolamine; I**_PC_**, ^+^N(CH_3_)_3_, phosphatidylcholine; I**_DGDG_**, CH-1”, digalactosyldiacylglycerol; I**_UNS_**, CH=CH of all unsaturated fatty acids.

**Figure 4:**
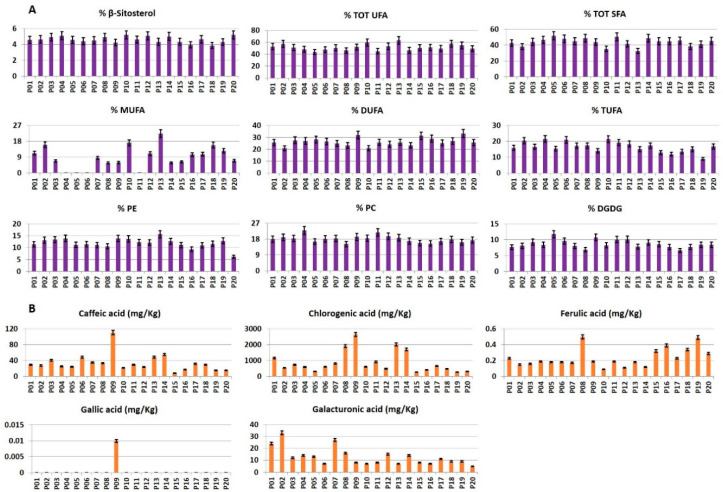
Histograms related to (**A**) compounds identified and quantified (molar % ± SD) in the ^1^H NMR spectra of organic Bligh–Dyer extracts and (**B**) compounds identified by RP-HPLC-PDA-ESI-MSn (Reversed Phase High-Performance Liquid Chromatography with Photodiode Array Detector and Electrospray Ionization Mass Detector) in hydroalcoholic extracts (mg/Kg dry weight ± SD).

In the case of potato organic extracts, no literature data on the ^1^H NMR assignment has been reported so far. The ^1^H NMR assignment, see Supplementary Table S3 and Figure 3, was carried out using 2D NMR experiments, spiking experiments and literature data regarding the ^1^H assignment of organic extracts from other vegetable matrices in the same experimental conditions [26,27]. β-Sitosterol, fatty acids, phosphatidylcholine, phosphatidylethanolamine and digalactosyldiacylglycerol were identified and quantified, see Figure 4A, in all the analyzed samples. The NMR identification of the most characteristic lipophilic metabolites is here discussed. The most characteristics signals of glycerogalactolipids and glycerophospholipids belongs to their head groups. In particular, the presence of digalactosyldiacylglycerol (DGDG) was suggested by the characteristic doublet (*J* = 3.8 Hz) at 4.87 ppm due to the equatorial CH-1” proton, that is typical of the external galactose ring in DGDG. The diagnostic spin system detected by ^1^H-^1^H TOCSY experiment allowed to identify the other protons of this sugar, namely CH-2” (3.76 ppm), CH-3” (3.70 ppm), CH-4” (3.91 ppm), CH-5” (3.70 ppm), and CH_2_-6” (3.73 and 3.81 ppm). Analogously, CH-1’ proton of internal DGDG galactose ring was also detected at 4.19 ppm, together with CH-2’, CH-3’, and CH-4’ protons (3.49, 3.47, and 3.88 ppm, respectively). Phosphatidylcholine was recognized by the characteristic singlet at 3.21 ppm due to methyl protons of ^+^N(CH_3_)_3_ moiety. This signal showed in ^1^H-^13^C HMBC experiment a long-range correlation with carbon at 66.5 ppm, belonging to CH_2_N^+^ moiety. The diagnostic spin system of CH_2_N^+^ protons at 3.64 ppm, detected by ^1^H-^1^H TOCSY experiment, allowed to identify protons of CH_2_OP group at 4.31 ppm. Phosphatidylethanolamine was identified by the triplet signal of CH_2_-N group at 3.13 ppm, *J*-coupled (5 Hz) with CH_2_OP proton at 4.08 ppm.

β-Sitosterol was identified by its typical singlet at 0.66 ppm due to CH_3_-18 group, showing in ^1^H-^13^C HMBC experiment a long-range correlation with carbons at 40.2, 42.9 and 56.4 ppm of CH_2_-12, C-13 and CH-17, respectively. The identification was confirmed by spiking experiment. 

RP-HPLC-PDA-ESI-MSn targeted analysis was applied to detect and quantify, see Appendix SB, Supplementary Table S4 and Figure 4B, compounds commonly occurring in potatoes [28], namely caffeic, chlorogenic, ferulic, gallic, and galacturonic acids. 

The metabolite profile of the twenty potato cultivars will be discussed focusing on the specific compound classes (Figure 2 and Figure 4).

*Free amino acids*. As expected from literature data [11], asparagine turned out to be the main amino acid in all samples, followed by glutamine, aspartate, γ-aminobutyric acid (GABA), and valine. Proline, leucine, isoleucine, threonine, alanine, tyrosine, and phenylalanine generally showed a low concentration (below 100 mg/100 g). P10 cultivar was characterized by the highest amounts of asparagine, aspartate, and threonine and the lowest amount of proline. P04 cultivar showed the highest concentrations of valine, GABA, and proline; in particular, proline content was at least four times higher with respect to the other cultivars. Alanine and glutamine amounts were present in the highest levels in P09 cultivar, characterized also by the lowest content of aspartate. P15, P16, and P19 samples showed the highest content of phenylalanine, tyrosine, and isoleucine, respectively. P11 cultivar was characterized by the lowest amounts of leucine and alanine, whereas P06 had the lowest amounts of GABA and glutamine. The lowest concentrations of asparagine and phenylalanine were detected in P03 and P13, respectively (Figure 2A). 

*Sugars and polyols*. Glucose, fructose, and sucrose were the main sugars, whereas galactose and myo-inositol were present in lower concentration. Glucose and fructose content were correlated. In particular, P03 cultivar was characterized by the highest content of both sugars, whereas P06 sample showed the lowest level together with galactose. Conversely, P06 cultivar had a high sucrose content as well as P09 and P13 cultivars. P03 and P09 cultivars was characterized by the highest content of monosaccharides and disaccharides. P04 and P01 cultivars showed the highest contents of myo-inositol and galactose, respectively (Figure 2B). The low content of glucose was found in P04, P06, P08, and P17 cultivars, whereas the lowest total mono- and disaccharides amount was detected in P17 and P19 cultivars. 

*Organic acids*. Citric acid turned out to be the most abundant organic acid in all samples. Specifically, the highest content was detected in P20 cultivar, P06 and P10 showing a significant concentration as well. On the contrary, P01 showed the lowest citric acid concentration. The lowest amount of lactic acid was found in P18 (four to eight times lower with respect to all the other samples). Conversely, P18 cultivar was characterized by the highest content of formic acid and together with P08 and P20, P18 cultivars, of malic acid. P10 cultivar showed the lowest formic acid one. The highest content of fumaric acid was observed in P07, P04, and P08 cultivars, whereas the lowest one was found in P11 (Figure 2C). Galacturonic acid was observed in high concentrations in P02 cultivar whereas the lowest amount was found in P20 cultivar (Figure 4B). 

*Miscellaneous compounds*. The highest content of choline was present in P03, P04, P06, P12, P14, and P19 cultivars, whereas P01 showed the lowest one (Figure 2D). P10, P17, P18, and P19 cultivars were the richest in trigonelline. 

*β-Sitosterol*. The β-sitosterol value was quite similar in all the samples with the highest value in P20 cultivar and the lowest one in P18 (Figure 4A).

*Fatty acids*. According to literature, mono-unsaturated fatty acids (MUFA) were present in low concentration [14,15,16], with P13 cultivar showing the highest value. In the case of P04, P05, P06, and P11 cultivars, MUFA level was not reported since, due to their extremely low MUFA concentration, the error associated with the Equation (4) turned out to be higher than the MUFA value. The lowest concentration of di-unsaturated fatty acids (DUFA) was present in P02 cultivar, whereas the highest DUFA level was in P19 cultivar. Conversely, P19 cultivar had the lowest concentration of tri-unsaturated fatty acids (TUFA), whereas the highest concentration was found in P10 sample. The highest concentration of total saturated fatty acids (TOT SFA) was detected in P05 cultivar, whereas the lowest one was found in P13 (Figure 4A).

*Polar lipids*. A comparable content of phosphatidylcholine (PC), phosphatidylethanolamine (PE) and digalactosyldiacylglycerol (DGDG) was detected in all the analyzed samples. The highest content of PC was found in P04 cultivar, whereas the lowest one was found in P08 sample. P13 cultivar showed the highest PE content, whereas P16 had the lowest one. DGDG was present in the highest concentration in P05 cultivar, whereas the lowest DGDG amount was measured in P17 cultivar (Figure 4A).

*Phenolics*. The levels of caffeic, chlorogenic, ferulic, and gallic acids were reported in Figure 4B. The concentration of caffeic acid, a hydroxycinnamic acid, was very low except in P09 cultivar. The highest concentration of chlorogenic acid was observed in blue/purple colored potato cultivars i.e., P08, P09, P13, and P14. Conversely, chlorogenic acid content was the lowest in P15 and P19 cultivars. Ferulic acid was detected in relatively low concentration, with the maximum value found in P08 cultivar and the lowest in P10. Gallic acid was detected only in P09 cultivar. 

## 4. Discussion

The results obtained from NMR and RP-HPLC-PDA-ESI-MSn analyses showed that every local cultivar is characterized by a proper chemical profile. As expected, Rouge De Flanders, Bergerac, Fleur Bleu, and Blue Star cultivars, characterized by blue/purple pulp and peel, showed high contents of chlorogenic acid, a bioactive compound with a strong antioxidant activity. In fact, chlorogenic acid and anthocyanins (responsible for blue/purple color of vegetables) are closely connected in their biosynthesis pathways [29]. In vitro and in vivo experimental studies have shown that chlorogenic acid and derivatives could influence glucose metabolism by lowering postprandial concentration in blood [30], stimulating insulin secretion and glucose uptake in muscle cells [31,32]. Owing to their high concentrations of chlorogenic acid and low content of mono- and disaccharides, Agria and Rouge des Flandres cultivars could be consumed by the subjects with dietary regimens where sugar intake has to be reduced and carbohydrate catabolism increased. Regarding sugar content, Jelly cultivar was characterized by high levels of monosaccharides. Agria cultivar was also characterized, together with Roseval and Rubra Spes, by high levels of citric acid, an organic acid involved in the inhibition of the enzymatic browning in fresh-cut potato [33]. Quarantina Bianca and Giana Riunda cultivars resulted to be the richest in the essential amino acids i.e., leucine, isoleucine, valine, threonine, and phenylalanine.

Glutamine together with asparagine and proline is involved in the Maillard reaction during potato cooking [11,12], generating volatile compounds responsible for the particular and pleasant smell of cooked potatoes. High glutamine contents were detected in Bergerac, Rouge des Flandres, Blue Star, and Roseval cultivars. Roseval cultivar also showed a high level of asparagine, whereas Ratte showed a very high proline content.

**Figure 5:**
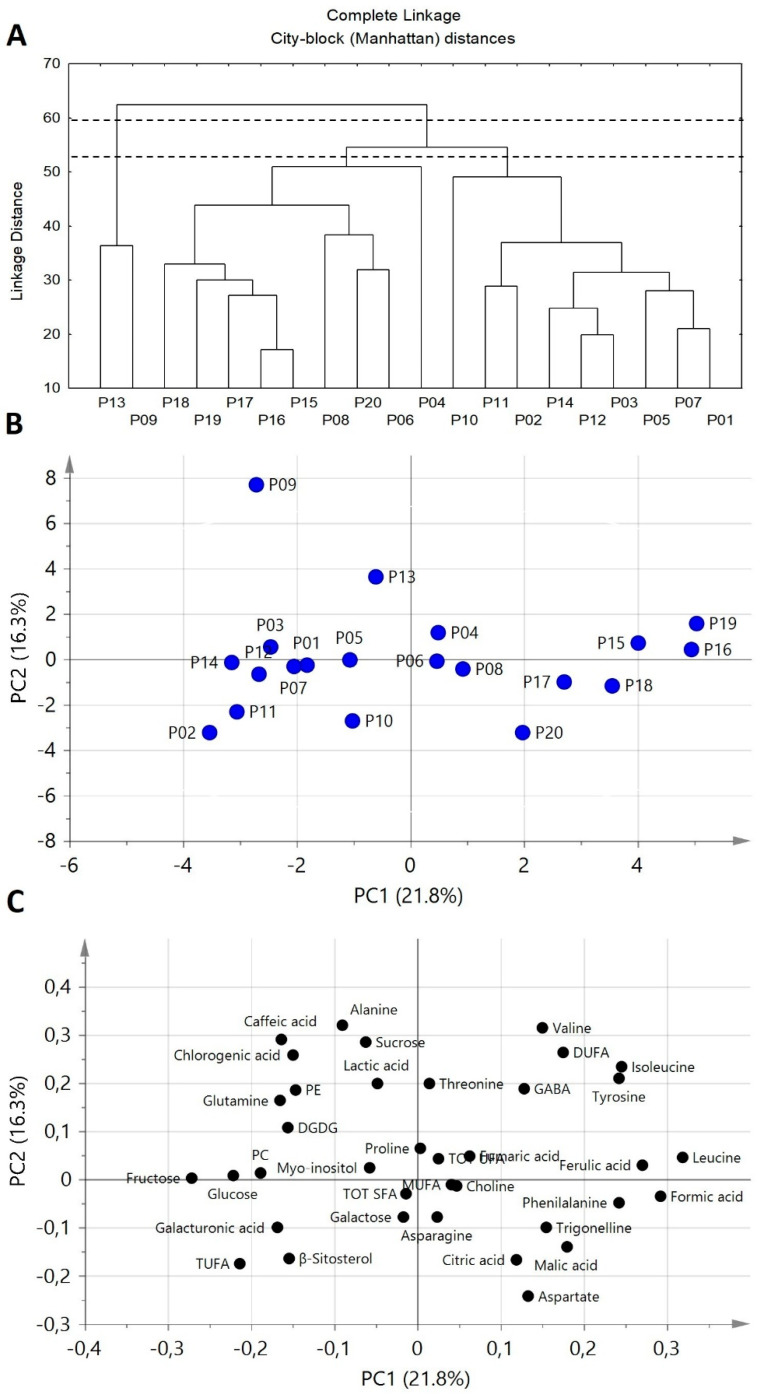
TCA (Tree Clustering Analysis) and PCA (Principal Component Analysis) applied to NMR and HPLC data relative to twenty potato cultivars (see Material and Methods section). Dendrogram (TCA) cut on two levels (**A**). PCA maps of potato sample scores (**B**) and loadings (**C**). PC1 and PC2 represent 21.8% and 16.3% of the total variance, respectively.

A comprehensive picture of chemical differences between potato cultivar is given by the explorative multivariate statistical analyses namely tree clustering analysis (TCA) and principal component analysis (PCA), see Figure 5, applied to NMR and HPLC data. TCA dendrogram allows similarities and differences between the different cultivars to be observed (Figure 5A). Cutting the dendrogram at a proper level, P13 and P09 turned out to be well distant from all the other samples, thus suggesting the peculiarity of P13 and P09 metabolite profiles with respect to other cultivar profiles. Cutting the dendrogram at a lower level, all remaining samples can be divided in two clusters: the first one includes P04, P06, P08, P15, P16, P17, P18, P19, and P20 cultivars, whereas the second one includes P01, P02, P03, P05, P07, P10, P11, P12, and P14 samples. 

In order to have information regarding the metabolites responsible for similarities/differences between cultivars, a PCA was also carried out. The scatter plot (Figure 5B) and the loading plot (Figure 5C) showed that P09 and P13 cultivars are characterized by high contents of chlorogenic acid and sucrose and low amounts of phenylalanine. P04, P06, P08, P15, P16, P17, P18, P19, and P20 cultivars cluster (positive PC1 scores) is characterized by high contents of leucine, isoleucine, phenylalanine, DUFA, and ferulic acid, and low levels of sugars. Conversely, the other cultivars (negative PC1 scores, except P13 and P09) were characterized by high levels of glucose and fructose.

## 5. Conclusions

Potatoes are important components of human diet as they are rich in nutrients and bioactive components useful to maintain a good nutritional status and wellness. The in-depth chemical composition study of twenty potato cultivars, here reported, can contribute to their valorization and introduction in national or international market as well as to their introduction as row material in food industries. The use of both untargeted and targeted methodologies showed to be a suitable strategy for a complete chemical characterization of food matrices, such as local potato cultivars in this case. In particular, the use of the untargeted NMR methodology allowed to have a complete chemical profile of the analyzed samples, identifying different classes of compounds namely sugars, amino acids, organic acids, fatty acids, polar lipids, and other metabolites. On the other hand, the targeted RP-HPLC-PDA-ESI-MSn methodology represented an effective strategy to measure specific compounds whose identification is designed a priori.

It is important that the chemical composition of this foodstuff is preserved during industrial processing, so a future study on the effect of industrial practices on the chemical composition of these potatoes cultivar is in progress.

## Supplementary Materials

The following are available online at https://www.mdpi.com/2304-8158/9/9/1216/s1, Appendix-SA. Plot design of potato cultivation. Appendix-SB. MS (Mass spectrometer) identification and calibration curves of caffeic, chlorogenic, ferulic, gallic and galacturonic acids. Table S1. Compounds and relative signals (^1^H NMR (protonic NMR) chemical shift, ppm) selected for the quantitative analysis of the hydroalcoholic and organic extracts by mean NMR. Figure S1. ^1^H-^1^H TOCSY (Total Correlated Spectroscopy) spectra (28 °C) of potato hydroalcoholic extract in phosphate buffer in D_2_O (pH 7.4) (0.5–8.0 ppm region is here considered). Figure S2. ^1^H-^13^C HSQC (Heteronuclear Single Quantum Correlation) spectra (28 °C) of potato hydroalcoholic extract in phosphate buffer in D_2_O (pH 7.4) (0.5–8.0 ppm region is here considered). Figure S3. ^1^H-^13^C HMBC (Heteronuclear Multiple Bond Correlation) spectra (28 °C) of potato hydroalcoholic extract in phosphate buffer in D_2_O (pH 7.4; 0.5–8.0 ppm region is here considered). Figure S4. ^1^H-^1^H TOCSY spectra (28 °C) of potato organic extract in CDCl_3_/CD_3_OD 2:1 *v/v* mixture (0–6.0 ppm region is here considered). Figure S5. ^1^H-^13^C HSQC spectra (28 °C) of potato organic extract in CDCl_3_/CD_3_OD 2:1 *v/v* mixture (0–6.0 ppm region is here considered). Figure S6. ^1^H-^13^C HMBC spectra (28 °C) of potato organic extract in CDCl_3_/CD_3_OD 2:1 *v/v* mixture (0–6.0 ppm region is here considered). Table S2: Metabolites identified in the 600.13 MHz ^1^H NMR spectra (28 °C) of potato hydroalcoholic extracts in phosphate buffer in D_2_O (pH 7.4). Table S3: Metabolites identified in the 600.13 MHz ^1^H NMR spectra (28 °C) of potato organic extracts in CDCl_3_/CD_3_OD (2:1 *v/v*) mixture. Table S4. Compounds identified with RP-HPLC-PDA-ESI-MSn (Reversed Phase High-Performance Liquid Chromatography with Photodiode Array Detector and Electrospray Ionization Mass Detector) analysis. The respective retention time, ʎ Max, *m/z* and fragment are reported.
