# Supplementary material for: Characterization of Local Products for Their Industrial Use: The Case of Italian Potato Cultivars Analyzed by Untargeted and Targeted Methodologies
Source: Foods. 2020 Sep 2;9(9):1216. doi: 10.3390/foods9091216 (PMC7555304; doi:10.3390/foods9091216)
Supplement: Supplementary file 1 [file foods-09-01216-s001.pdf]

Characterization of local products for their industrial use: the case of Italian potato cultivars analyzed by untargeted and targeted methodologies

Cinzia Ingallina[1ξ], Mattia Spano[1ξ], Anatoly P. Sobolev[2*], Cristina Esposito[3], Cristina Santarcangelo[3], Alessandra Baldi[4], Maria Daglia[3,5*], Luisa Mannina[1,2]

[1] Department of Chemistry and Technology of Drugs, Sapienza University of Rome, Piazzale Aldo Moro 5, 00185 Rome, Italy; cinzia.ingallina@uniroma1.it (C.I.); mattia.spano@uniroma1.it (M.S.); luisa.mannina@uniroma1.it (L.M.)
[2] Institute for Biological Systems, Magnetic Resonance Laboratory "Segre-Capitani", CNR, Via Salaria Km 29.300, 00015 Monterotondo (Rome), Italy
[3] Department of Pharmacy, University of Naples Federico II, Naples, 80138, Italy; cristina.esposito@unina.it. (C.E.); cristina.santarcangelo@unina.it (C.S.)
[4] Tefarco Innova, Parco Area delle Scienze 27/A – Campus 43124 Parma, Italy; alessandra.baldi.alimenti@gmail.com (A.B.)
[5] International Research Center for Food Nutrition and Safety, Jiangsu University, 212013 Zhenjiang, China
* Correspondence: anatoly.sobolev@cnr.it (A.P.S.); maria.daglia@unina.it (M.D.); Tel.: +39-06-9067-2385 (A.P.S.); Tel.: +39-081-678644

[ξ]These authors contributed equally to this work

Received: date; Accepted: date; Published: date

Appendix-SA. Plot design of potato cultivation.

For each cultivar, a block of 50 rows (50 tubers for row) was planted, with an 80 cm distance between tubers.

Appendix-SB. MS identification and calibration curves of caffeic, chlorogenic, ferulic, gallic and galacturonic acids.

App-B.1 MS identification of caffeic, chlorogenic, ferulic, gallic and galacturonic acids

Caffeic acid showed parent ion with *m/z* of 179 and principal fragment at *m/z* of 135, which derived by the CO_2 loss (- 44 Da) from the carboxylic acid group. Chlorogenic acid presented a molecular ion at *m/z* 353 and fragmentation ions 191, 179, 135. Ferulic acid showed [M-H]− m/z of 193 and a fragment ion at *m/z* 134 corresponding to $[M\text{-}CO_2\text{–}CH_3]^-$. Gallic acid and galacturonic acid present as parent ions with *m/z* of 169 and 193, respectively. Both compounds break up to CO_2 loss (- 44 Da) with production of fragment ions at *m/z* of 125 and 149, respectively.

App-B.2 Calibration curves of caffeic, chlorogenic, ferulic, gallic and galacturonic acids

To quantify caffeic, chlorogenic, ferulic, gallic and galacturonic acids, a calibration curve was constructed using external standards for each compound. Stock solutions of analytical standard grade caffeic, chlorogenic, ferulic, gallic and galacturonic acids (10 µg/mL) were prepared in methanol. Caffeic and chlorogenic acids stock solutions were diluted to obtain five concentrations ranging from 10 to 1000 ng/mL ($R^2 = 0.9998$). Ferulic, gallic and galacturonic acids stock solutions were diluted to obtain five concentrations ranging from 0.01 to 50 ng/mL ($R^2 = 0.9996$). The analyses were performed in triplicate for each concentration.

Table S1. Compounds and relative signals (^1H NMR chemical shift, ppm) selected for the quantitative analysis of the hydroalcoholic and organic extracts by mean NMR.

^1H Chemical shift (ppm)	Group	Compound
**Hydroalcoholic extract**		
0.96	CH_3	Leucine
1.02	CH_3	Isoleucine
1.05	CH_3	Valine
1.33	CH_3	Lactic acid
1.34	CH_3	Threonine
1.49	CH_3	Alanine
2.01	CH_2	Proline
2.30	CH_2	GABA
2.46	CH	Glutamine
2.55	CH	Citric Acid
2.81	CH_2	Aspartate
2.97	CH_2	Asparagine
3.21	$^+N(CH_3)_3$	Choline
3.30	CH	Myo-inositol
4.12	CH	Fructose
4.31	CH	Malic Acid
4.60	CH	β-Galactose
4.66	CH	β-Glucose
5.25	CH	α-Glucose
5.28	CH	α-Galactose
5.42	CH	Sucrose
6.53	CH	Fumaric acid
6.90	CH	Tyrosine
7.43	CH	Phenylalanine
8.46	CH	Formic Acid
8.84	CH	Trigonelline
**Organic extract**		
0.66	CH_3	β-Sitosterol
2.30	α-CH_2	Total fatty acids
2.73	CH_2	Di-unsaturated fatty acids
2.77	CH_2	Tri-unsaturated fatty acids
3.12	CH_2	Phosphatidylethanolamine
3.21	$^+N(CH_3)_3$	Phosphatidylcholine
4.87	CH	Digalactosyldiacylglycerol
5.31	CH=CH	Total unsaturated fatty acids

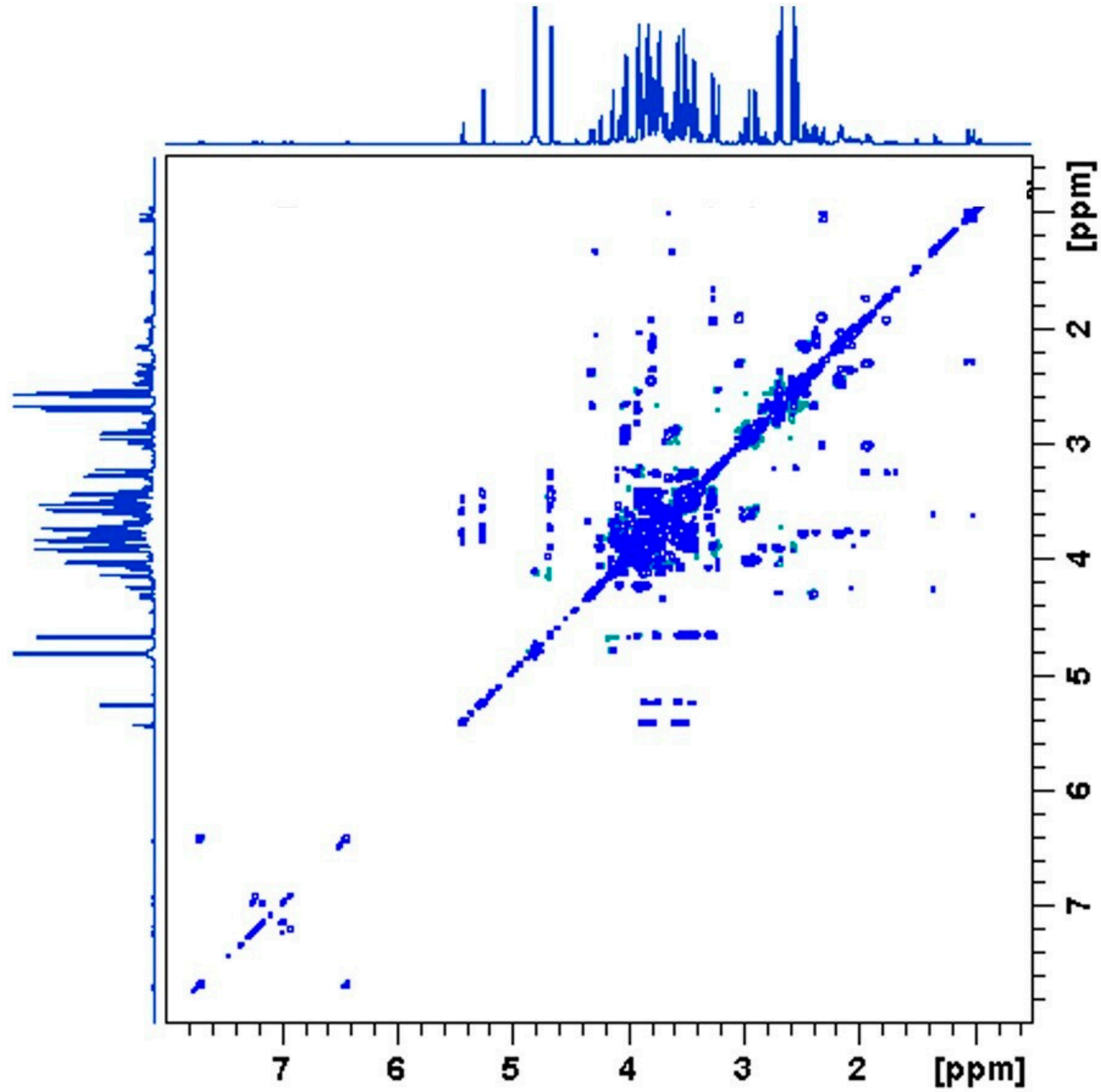

Figure S1. ^{1}H-^{1}H TOCSY spectra (28 °C) of potato hydroalcoholic extract in 400 mM phosphate buffer in D_2O (pH 7.4) 1 mM TSP (0.5 – 8.0 ppm region is here considered).

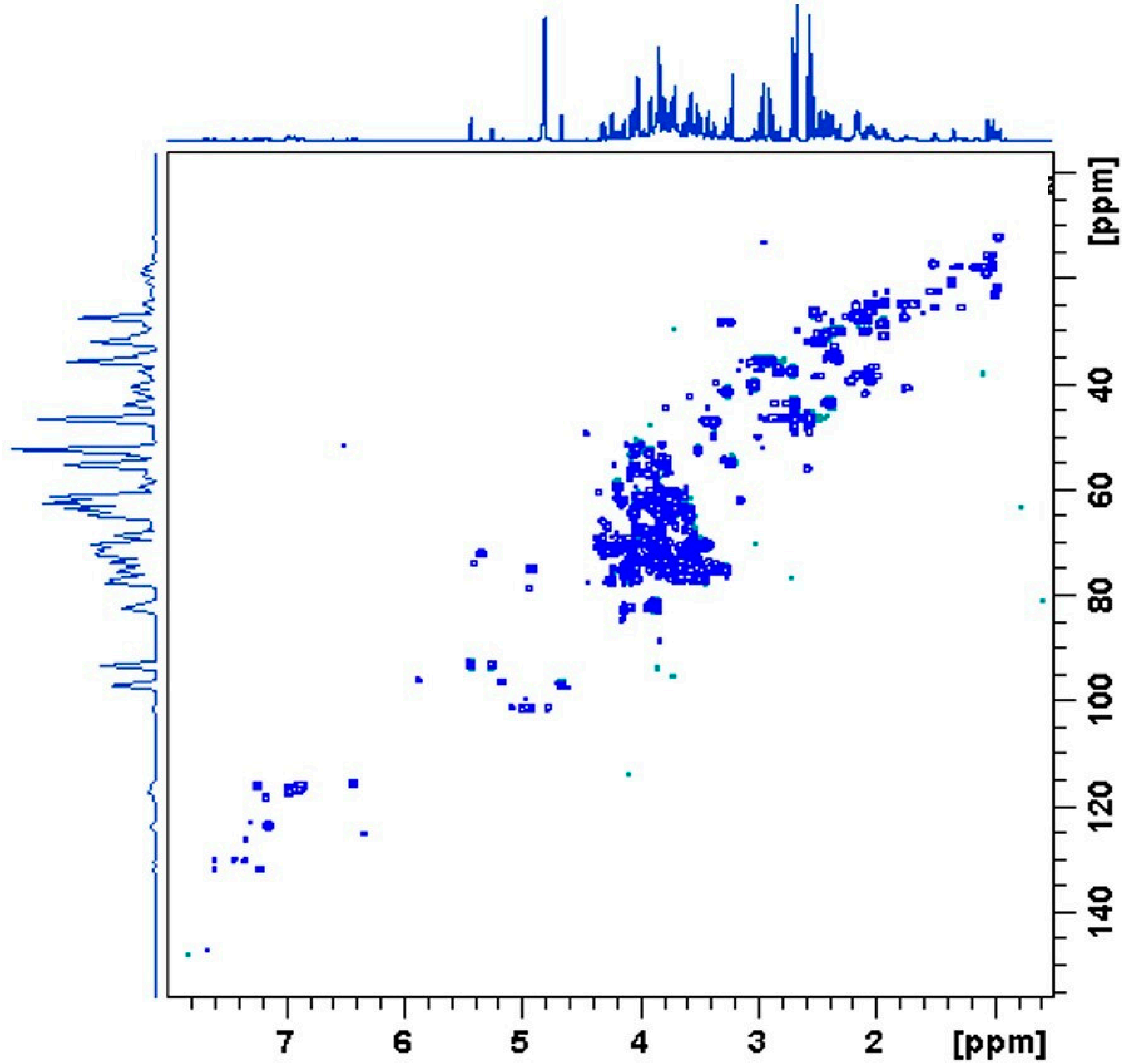

Figure S2. ^{1}H-^{13}C HSQC spectra (28 °C) of potato hydroalcoholic extract in phosphate buffer in D_2O (pH 7.4) (0.5 – 8.0 ppm region is here considered).

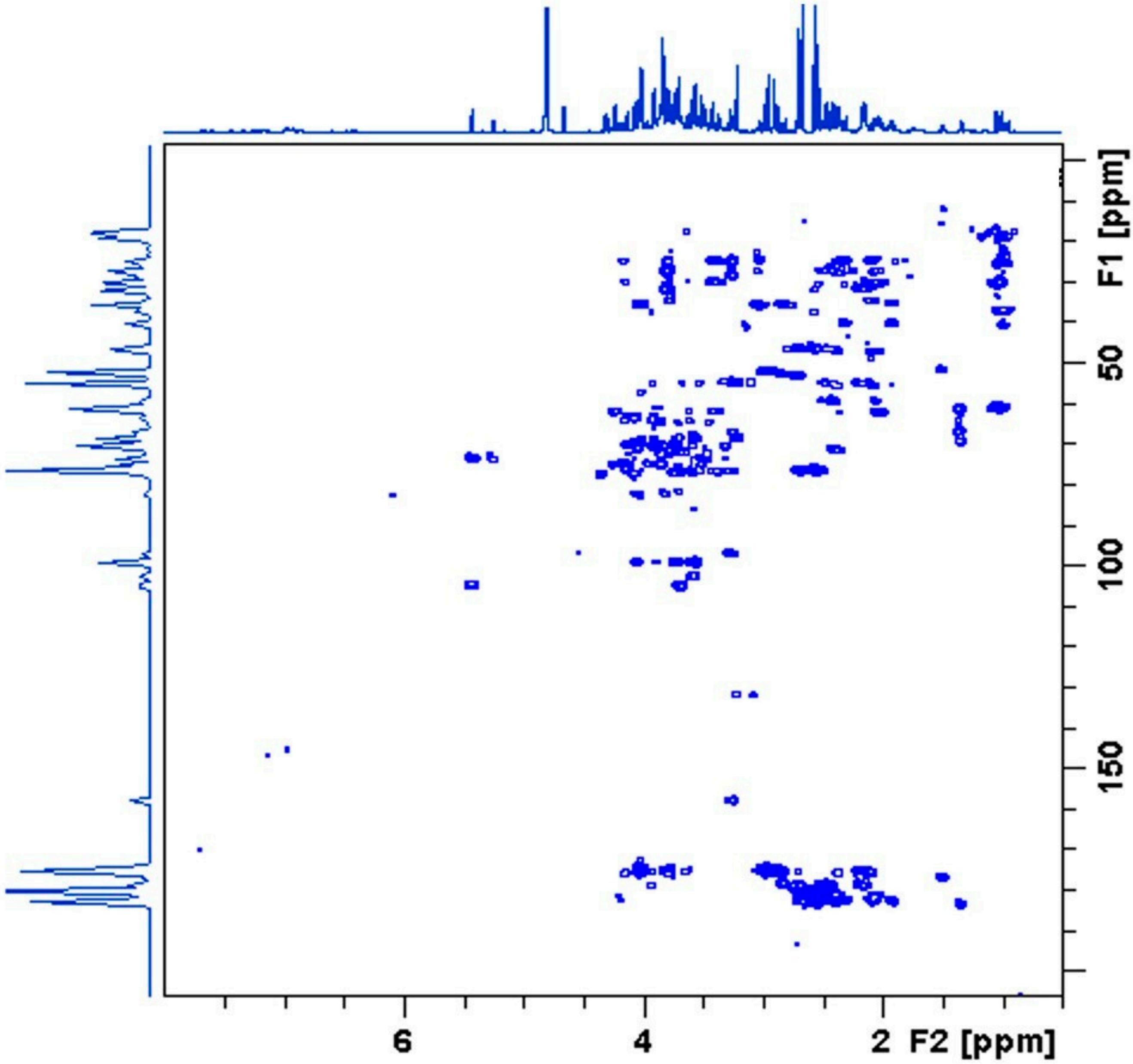

Figure S3. ^{1}H-^{13}C HMBC spectra (28 °C) of potato hydroalcoholic extract in phosphate buffer in D_2O (pH 7.4) (0.5 – 8.0 ppm region is here considered).

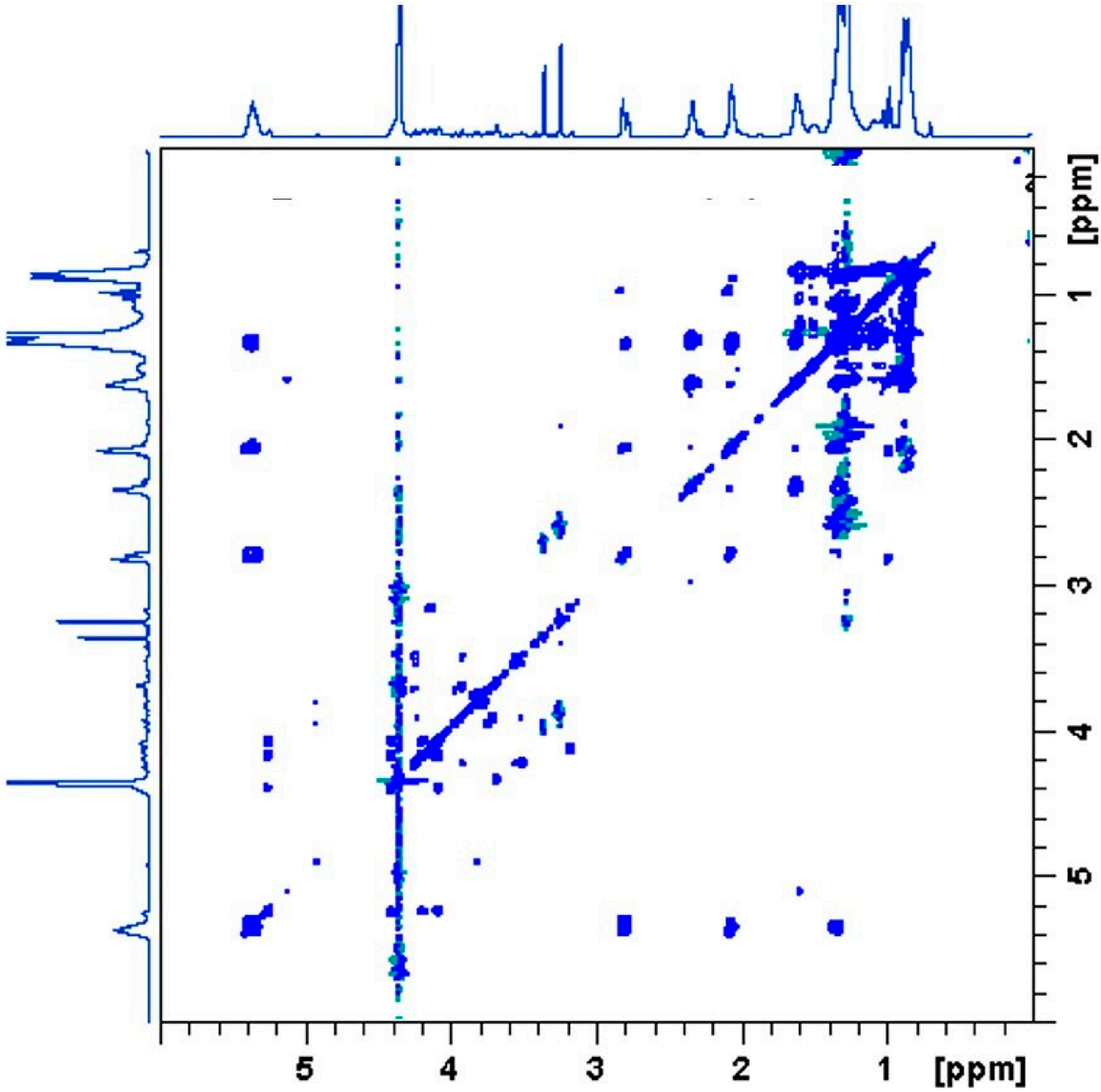

Figure S4. ^{1}H-^{1}H TOCSY spectra (28 °C) of potato organic extract in $CDCl_3/CD_3OD$ 2:1 *v/v* mixture (0 – 6.0 ppm region is here considered).

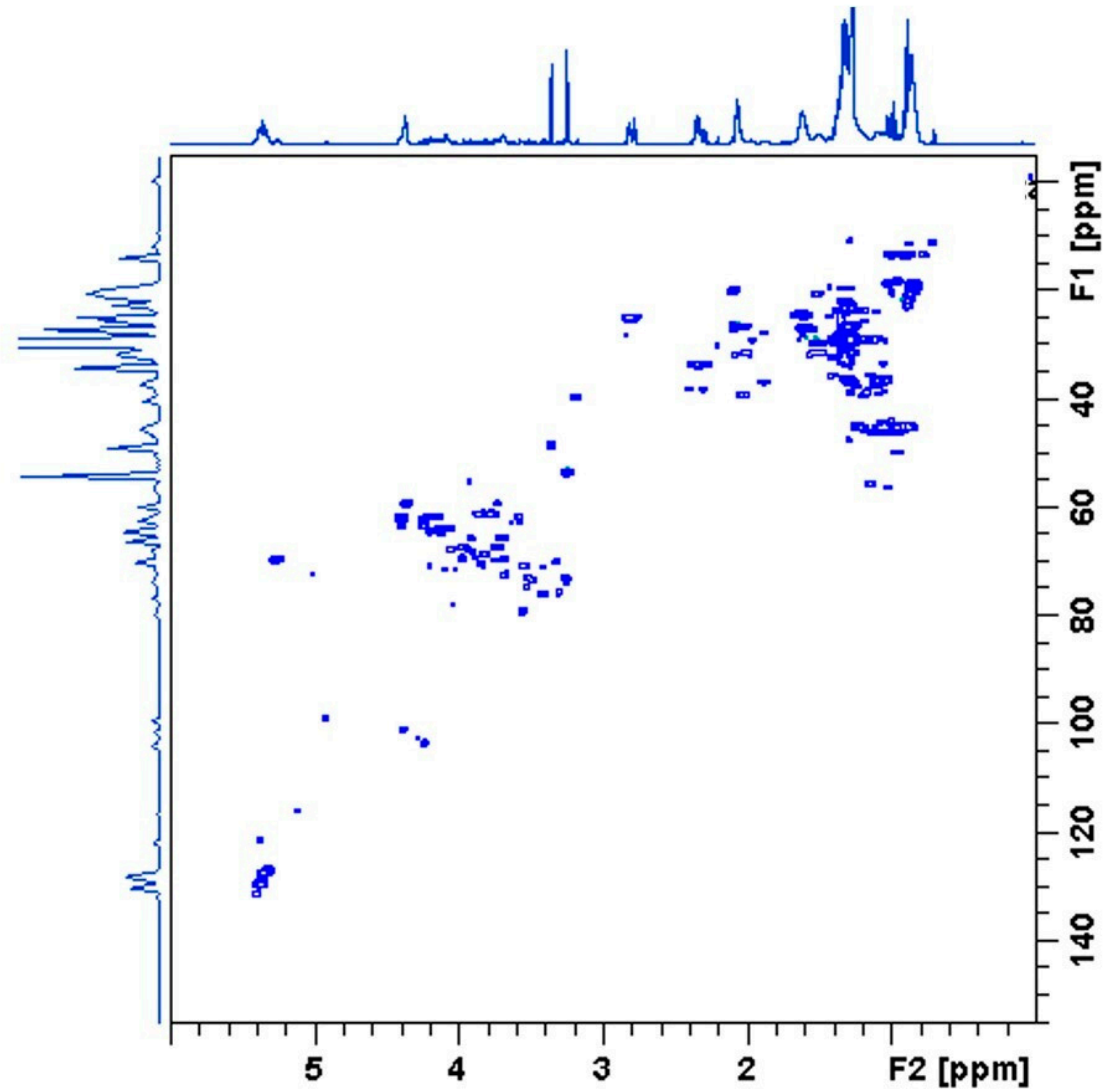

Figure S5. ^{1}H-^{13}C HSQC spectra (28 °C) of potato organic extract in $CDCl_3/CD_3OD$ 2:1 *v/v* mixture (0 – 6.0 ppm region is here considered).

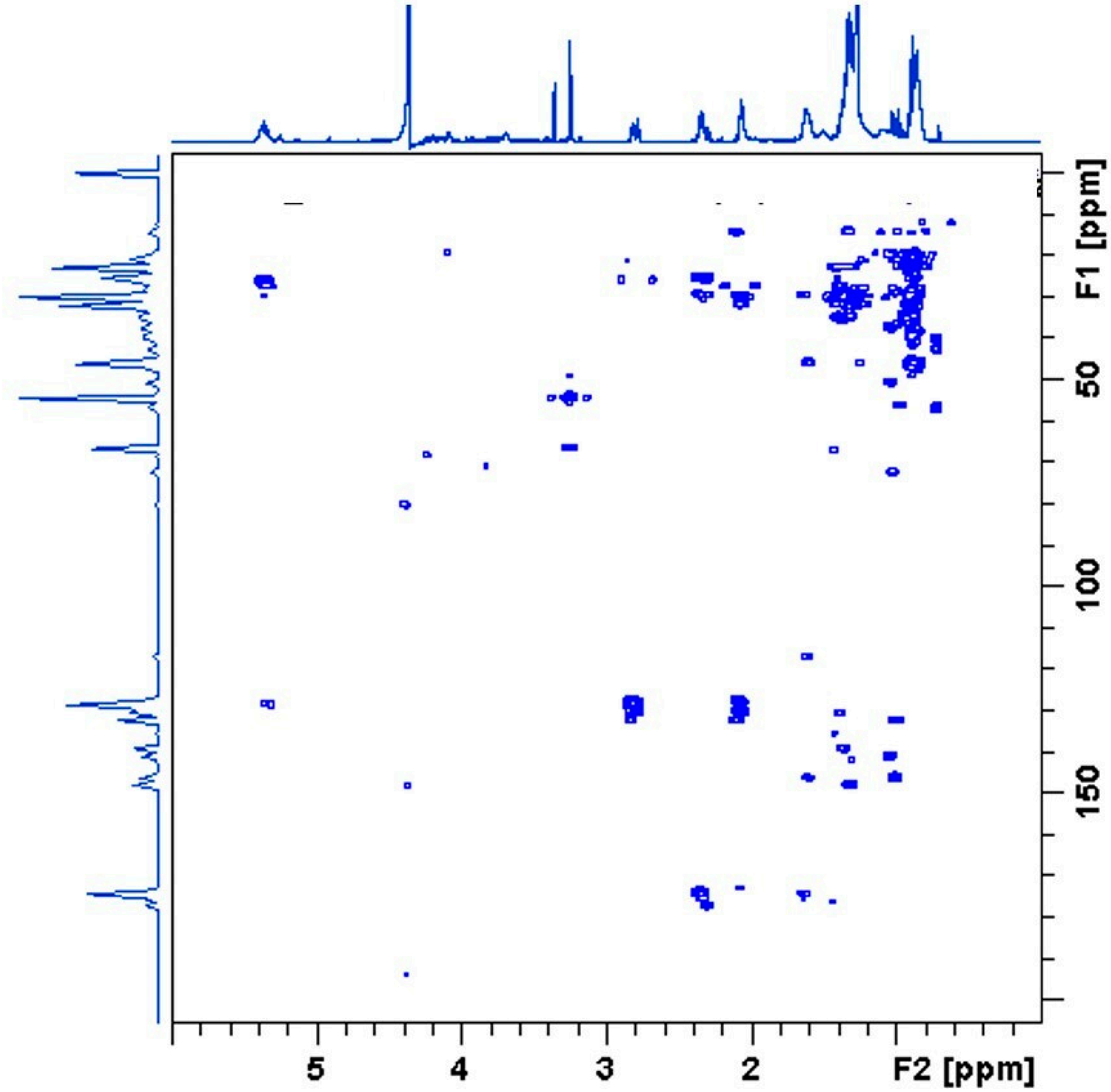

Figure S6. ^{1}H-^{13}C HMBC spectra (28 °C) of potato organic extract in $CDCl_3$/CD_3OD 2:1 *v/v* mixture (0 – 6.0 ppm region is here considered).

Table S2. Metabolites identified in the 600.13 MHz ^1H NMR spectra (28 °C) of potato hydroalcoholic extracts in phosphate buffer in D_2O (pH 7.4).

Compound	Assignment	^1H (ppm)	Multiplicity [J(Hz)]	^{13}C (ppm)
**Sugars and polyols**				
α-D-Fructofuranose	CH-3	4.12		83.0
	CH-5	4.07		82.5
β-D-Fructofuranose	CH-3	4.12		76.5
	CH-4	4.12		75.4
	CH-5	3.81		
	CH_2-6,6'	3.70, 3.84		
β-D-Fructopyranose	CH-3	3.80		68.6
	CH-4	4.00		77.0
	CH-5	4.04		
	CH_2-6,6'	3.71; 4.08		64.4
α-Galactose	CH-1	5.28	d [3.8]	92.3
	CH-2	3.87		
	CH-3	3.99		
β-Galactose	CH-1	4.60	d [8.0]	97.1
	CH-2	3.51		
	CH-3	3.67		
	CH-4	3.95		
α-Glucose	CH-1	5.25	d [3.8]	93.2
	CH-2	3.54		72.0
	CH-3	3.71		73.3
	CH-4	3.42		70.7
	CH-5	3.84		72.5
	CH_2-6,6'	3.89; 3.75		61.8
β-Glucose	CH-1	4.66	d [8.0]	97.0
	CH-2	3.26		75.1
	CH-3	3.51		76.9
	CH-4	3.42		70.7

	CH-5	3.49		76.8
	CH_2-6,6'	3.90; 3.75		61.8
Myo-Inositol	CH-1	4.07		
	CH-2,5	3.57		
	CH-3,6	3.65		
	CH-4	3.30	t [9.5]	74.2
Sucrose	CH-1 (Glucose)	5.42	d [3.8]	93.3
	CH-2	3.58		71.8
	CH-3	3.78		73.6
	CH-4	3.50		70.2
	CH-5	3.86		73.5
	CH-3' (Fructose)	4.22	d [8.7]	77.5
	CH-4'	4.07		75.1
	CH-5'	3.90		82.4
	CH_2-6'	3.83		61.2
**Organic acids**				
Citric acid	α,γ-CH	2.55	d [15.9]	46.6
	α',γ'-CH	2.68		46.6
	β-C			76.4
	1,5-COOH			180.2
	6-COOH			183.0
Formic acid	HCOOH	8.46	s	
Fumaric Acid	α,β–CH=CH	6.52	s	
Lactic acid	β–CH_3	1.33	d [7.0]	20.6
	α-CH	4.12		69.3
	COOH			183.4
Malic acid	α-CH	4.31	dd [9.8; 3.2]	71.4
	β-CH	2.70	dd [15.6; 3.2]	43.9
	β'-CH	2.39	dd [15.6; 9.8]	43.9
**Amino acids**				

Alanine	α-CH	3.80		51.5
	β-CH_3	1.49	d [7.3]	17.3
	COOH			176.8
Arginine	α-CH	3.77		55.3
	β-CH_2	1.91	m	28.6
	γ-CH	1.67	m	25.1
	γ'-CH	1.74	m	25.1
	δ-CH_3	3.24		41.6
Asparagine	α-CH	4.02		52.3
	β,β'-CH_2	2.89; 2.97	dd [7.4; 16.9]	35.8
Aspartate	α-CH	3.91		
	β,β'-CH_2	2.70; 2.81	dd [3.9; 17.4]	37.7
γ-Aminobutyrate	α-CH_2	2.30	t [7.4]	35.4
	β-CH_2	1.91		24.3
	γ-CH_2	3.01	t [7.6]	39.8
Glutamine	α-CH	3.79		54.5
	β,β'-CH_2	2.15	m	26.8
	γ-CH	2.46	m	32.2
Glutamate	α-CH	3.75		
	β,β'-CH_2	2.07	m	28.0
	γ-CH_2	2.35	m	34.6
Isoleucine	α-CH	3.69		
	β-CH	1.98		36.7
	γ-CH_3	1.27		25.1
	γ-CH_3	1.02	d [7.0]	15.7
	δ-CH_3	0.94		
Leucine	β-CH_2	1.74		
	γ-CH	1.71		
	δ-CH_3	0.97	d [6.3]	23.1
	δ'-CH_3	0.96	d [6.3]	22.1

Lysine	α-CH	3.74		
	β-CH_2	1.91	m	31.0
	γ-CH	1.45	m	22.6
	γ’-CH	1.52	m	22.6
	δ-CH_2	1.73	m	27.3
	ε-CH_2	3.02		
Phenylalanine	CH-2,6	7.34	m	
	CH-4	7.38	m	
	CH-3,5	7.43	m	130.2
Proline	β-CH	2.35		
	γ-CH_2	2.01	m	24.8
Threonine	α-CH	3.60		61.1
	β-CH	4.31		66.7
	γ-CH_3	1.34	d [6.6]	20.6
Tyrosine	CH-3,5	7.20	d [8.5]	131.9
	CH-2,6	6.90	d [8.5]	116.9
Valine	α-CH	3.62		60.6
	β–CH	2.28		29.7
	γ-CH_3	1.00	d [7.0]	17.9
	γ'-CH_3	1.05	d [7.0]	19.1
**Phenolics**				
Chlorogenic acid	α-CH=	6.42	d [16.0]	115.8
	β–CH=	7.68	d [16.0]	
	CH_2-2	2.20	m	
	CH-3	5.33	m	72.2
	CH-4	3.88		
	CH-5	4.26		71.6
	CH_2-6	2.04	m	
Caffeic acid	α-CH=	6.35	d [16.0]	
	β–CH=	7.29	d [16.0]	

**Miscellaneous metabolites**				
Choline	$^{+}N(CH_3)_3$	3.21	s	55.1
	α-CH_2	3.81		68.5
Trigonelline	CH-1	9.12	s	
	CH-3,5	8.84		
	CH-4	8.08		

Table S3. Metabolites identified in the 600.13 MHz ^{1}H NMR spectra (28 °C) of potato organic extracts in $CDCl_3/CD_3OD$ 2:1 *v/v* mixture.

Compound	Assignment	^{1}H (ppm)	Multiplicity: *J* [Hz]	^{13}C (ppm)
Mono-unsaturated fatty chain	COO			174.4
(Cn:1 Δ^9)	CH_2-2	2.30		34.6
	CH_2-3	1.57	m	25.4
	CH_2-4,7	1.30	m	29.5
	CH_2-8	2.01	m	27.6
	CH=CH 9,10	5.30	m	130.4
	CH_2-11	2.01	m	27.6
	CH_2	1.33-1.28	m	29.6-31.7
	CH_2-n-1	1.26	m	22.9
	CH_3-n	0.84	t	14.2
Di-unsaturated fatty chain	COO			174.4
(Cn:2 $\Delta^{9,12}$)	CH_2-2	2.30		34.6
	CH_2-3	1.57	m	25.4
	CH_2-4,7	1.32-1.28	m	29.5
	CH_2-8	2.02	m	27.6
	CH= 9	5.32	m	130.4
	CH= 10	5.30	m	128.6
	CH_2-11	2.73	t [6.8]	26.0
	CH= 12	5.30	m	128.6
	CH= 13	5.32	m	130.4
	CH_2-14	2.02	m	27.6
	CH_2	1.26-1.27	m	29.4-31.7
	CH_2-n-1	1.23	m	22.9

	CH_3-n	0.85	t	14.2
Linolenic fatty chain (C18:3 $\Delta^{9,12,15}$)	COO			174.4
	CH_2-2	2.30		34.6
	CH_2-3	1.57	m	25.4
	CH_2-4,7	1.30	m	29.5
	CH_2-8	2.03	m	27.6
	CH= 9	5.32	m	130.4
	CH= 10	5.30	m	128.6
	CH_2 11	2.77	t [6.2]	26.0
	CH=CH 12,13	5.30	m	128.6
	CH_2-14	2.77	t [6.2]	26.0
	CH= 15	5.27	m	127.5
	CH= 16	5.34	m	132.2
	CH_2-17	2.03	m	20.9
	CH_3-18	0.94	t [7.6]	14.4
Saturated fatty acids	COO			174.4
	CH_2-2	2.28		34.6
	CH_2-3	1.57	m	25.4
	CH_2	1.28-1.22	m	29.6-32.0
	CH_2 n-1	1.25		23.0
	CH_3 n	0.84	t	14.2
β-Sitosterol	CH_2-1	1.83; 1.04		37.3
	CH-3	3.51		71.6
	CH-6	5.33		122.3
	CH_2-7	1.96; 1.47		32.3
	CH-8	1.44		32.4
	CH-9	0.90		50.6
	CH_2-11	1.47		21.4
	CH_2-12	1.99; 1.14		40.2
	CH-14	0.97		57.2
	C-13			42.9
	CH_2-15	1.55; 1.05		24.7
	CH_2-16	1.83; 1.25		28.6
	CH-17	1.09		56.4
	CH_3-18	0.66	s	12.1
	CH_3-19	0.97		19.5

	CH-20	1.36		36.5
	CH_3-21	0.90		19.1
	CH_2-22	1.27; 1.00		34.5
	CH_2-23	1.14		26.5
	CH-24	0.90		46.1
	CH_3-26	0.80		20.0
	CH_3-27	0.79		19.2
	CH_2-28	1.26		22.9
	CH_3-29	0.81		12.2
1,2-Diacyl-*sn*-glycero-3-phosphatidylethanolamine	CH_2N	3.13	t [5.0]	40.6
	CH_2OP	4.08		62.5
	CH_2 *sn1*	4.35; 4.13		62.6
	CH *sn2*	5.20		70.4
	CH_2 *sn3*	4.04		64.7
1,2-Diacyl-*sn*-glycero-3-phosphatidylcholine	$^+N(CH_3)_3$	3.21	s	54.5
	CH_2N^+	3.64		66.5
	CH_2OP	4.31		60.1
	CH_2 *sn1*	4.35; 4.13		62.6
	CH *sn2*	5.20		70.4
	CH_2 *sn3*	4.04		64.7
Digalactosyldiacylglycerol	CH_2 *sn1*	4.34; 4.19		63.2
	CH *sn2*	5.22		70.6
	CH_2 *sn3*	3.91; 3.67		68.1
	CH-1′′	4.87	d [3.8]	99.7
	CH-2′′	3.76		69.4
	CH-3′′; CH-5′′	3.70		70.6
	CH-4′′	3.91		70.2
	CH_2-6′′	3.81; 3.73		62.1
	CH-1′	4.19		104.3
	CH-2′	3.49		71.6
	CH-3′	3.47		73.7
	CH-4′	3.88		68.6

Table S4. Compounds identified with RP-HPLC-PDA-ESI-MSn analysis. The respective retention time, ʎ Max, *m/z* and fragment are reported.

**Compound**	**Retention time (min)**	**ʎ Max (nm)**	**m/z [M-H]$^-$**	**Fragment**
Caffeic acid	0.56	295, 325	179	135 (100)
Chlorogenic acid	1.10	262, 321	353	191 (100), 179 (80), 135 (20)
Gallic acid	2.63	221, 269	169	125 (100)
Ferulic acid	2.93	236, 322	193	134 (100)
Galacturonic acid	4.03	208	193	149 (100)
